# Circulating miR-3659 may be a potential biomarker of dyslipidemia in patients with obesity

**DOI:** 10.1186/s12967-019-1776-8

**Published:** 2019-01-14

**Authors:** Liu Miao, Rui-Xing Yin, Shang-Ling Pan, Shuo Yang, De-Zhai Yang, Wei-Xiong Lin

**Affiliations:** 1 Department of Cardiology, Institute of Cardiovascular Diseases, The First Affiliated Hospital, Guangxi Medical University, Nanning, 530021 Guangxi People’s Republic of China; 2 Department of Pathophysiology, School of Premedical Science, Guangxi Medical University, Nanning, 530021 Guangxi People’s Republic of China; 3 Department of Molecular Genetics, Medical Scientific Research Center, Guangxi Medical University, Nanning, 530021 Guangxi People’s Republic of China

**Keywords:** Gene Expression Omnibus, Weighted gene co-expression network analysis, Dyslipidemia, Obesity, COL1A1, miR-3659

## Abstract

**Background:**

The present study attempted to identify potential key genes and miRNAs of dyslipidemia in obese, and to investigate the possible mechanisms associated with them.

**Methods:**

The microarray data of GSE66676 were downloaded, including 67 obese samples from the Gene Expression Omnibus (GEO) database. The weighted gene co-expression network (WGCNA) analysis was performed using WGCNA package and grey60 module was considered as the highest correlation. Gene Ontology annotation and the Kyoto Encyclopedia of Genes and Genomes (KEGG) pathway enrichment analyses for this module were performed by clusterProfiler and DOSE package. A protein–protein interaction (PPI) network was established using Cytoscape software, and significant modules were analyzed using molecular complex detection.

**Results:**

Collagen type I alpha 1 chain gene (*COL1A1*) had the best significant meaning. After bioinformatic analysis, we identified four miRNAs (hsa-miR-3659, hsa-miR-4658, hsa-miR151a-5p and hsa-miR-151b) which can bind SNPs in 3′UTR in *COL1A1*. After validation with RT-qPCR, only two miRNAs (hsa-miR-3659 and hsa-miR151a-5p) had statistical significance.

**Conclusions:**

The area of 0.806 for miR-3659 and 0.769 for miR-151a-5p under the ROC curve (AUC) may have good diagnostic value for dyslipidemia. Circulating miR-3659 may be a potential biomarker of dyslipidemia in patients with obesity.

**Electronic supplementary material:**

The online version of this article (10.1186/s12967-019-1776-8) contains supplementary material, which is available to authorized users.

## Background

Obesity, especially abdominal obesity, is the key reason to result in metabolic syndrome (MetS), which refers to insulin resistance, type 2 diabetes mellitus, hypertension, and dyslipidemia, and all above risk factors finally lead to cardiovascular disease [1, 2]. A recent study showed that about 2.2 billion people were overweight or obese in 2015 [3]. As a complex and multifactorial disease, lots of environmental and genetic factors can result in this disorder [4, 5].

MicroRNA (miRNA), a class of non-coding RNA molecules (~ 22 nucleotides), is short and highly conserved. When it dysregulated, lots of human diseases would be happened [6]. MiRNAs mediate post-transcriptional regulation of protein-coding genes by complementary binding to the 3′ untranslated region (3′UTR) and occasionally to the 5′UTR or coding regions of target mRNAs [7]. Previous study has shown that single nucleotide polymorphisms (SNPs) in the miRNA regulatory networks were a novel class of functional variants in the human genome. Genetic variants that potentially influence miRNA-mediated cellular function may be classified in two major categories: SNPs affecting miRNA biogenesis and SNPs in the miRNA targetome [8, 9].

Early detection and treatment contribute to a promising effect. Since the discovery of circulating miRNAs in body fluids, an increasing number of studies have focused on their potential and non-invasive biomarkers, and therapeutic targets or tools for many diseases [10]. Martino et al. found that both miR-33a and miR-33b were early biomarkers for cholesterol levels in childhood [11]. Besides, Iacomino et al. had further found about the role of miRNAs in obesity and related metabolic abnormalities [12]. In this study, we performed the integrated bioinformatic methods to construct the co-expression network and mark significant miRNA. The outcomes may help us for further elucidating the innate character of dyslipidemia, and provide new insights to potential biomarkers and signaling pathways to treat dyslipidemia in obese.

## Materials and methods

### Microarray data

**Fig. 1:**
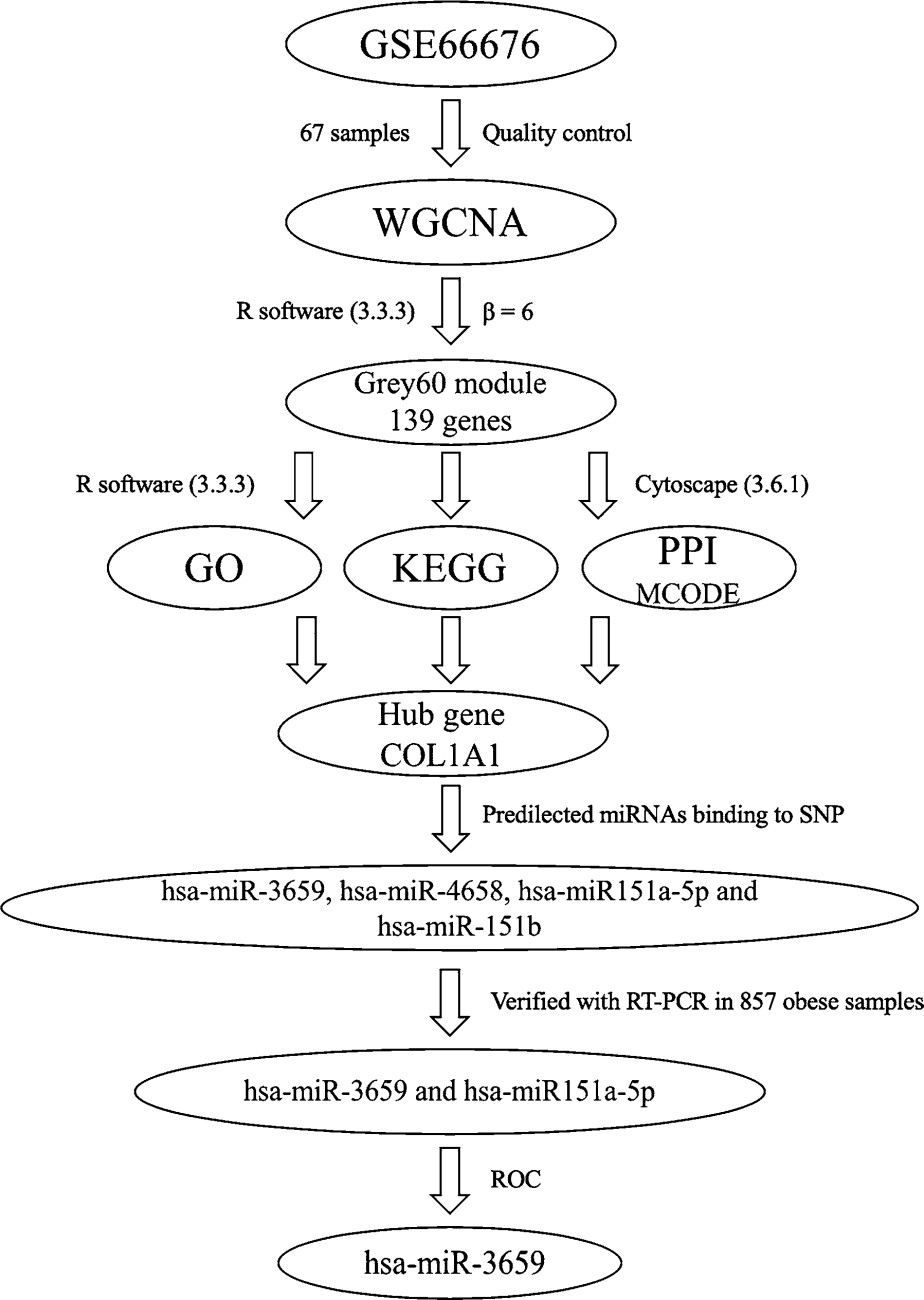
A flowchart for analysis. *GO* Gene Ontology annotation, *KEGG* the Kyoto Encyclopedia of Genes and Genomes pathway enrichment analyses, *PPI* protein–protein interaction, *MCODE* molecular complex detection

Microarray data of GSE66676 [13] were downloaded from the National Center for Biotechnology Information (NCBI) Gene Expression Omnibus (GEO, http://www.ncbi.nlm.nih.gov/geo/) database [14]. GSE66676 contains 67 Liver wedge biopsy samples from obesity patients (mean age = 16.88, mean body mass index, BMI = 52.01). The CEL files were transformed into the expression value matrix using the Affy package in R [15], and the probe information was then transformed into the gene name using Bioconductor in R [16]. If one gene had more than one probe, the mean expression value of this gene was selected. The specific workflow is shown in Fig. 1.

### Construction of weighted gene co-expression network

The weighted gene co-expression network (WGCNA) is a widely used systems biology method, which is used to construct a scale-free network from gene expression data [17]. An appropriate soft threshold power (soft power = 6) was selected in accordance with standard scale-free networks, with which adjacencies between all differential genes were calculated by a power function [18]. The rest of the analysis strategy can refer to our previous research [19].

### Finding module of interest and functional annotation

The correlation between modules and clinical features was evaluated by Pearson’s correlation tests to search biologically meaningful modules. The module and clinical feature, which exhibited the highest correlation, were selected as module of interest and clinical feature to be studied. All genes of module of interest were analyzed by Gene Ontology (GO) and Kyoto Encyclopedia of Genes and Genomes (KEGG) pathway by clusterProfiler and DOSE package in R [20]. A *P* value < 0.01 and false discovery rate (FDR) < 0.05 were set as the cutoff criteria.

### Hub gene analysis

The module membership (MM) was defined as the correlation of gene expression profile with module eigengenes (Mes). And the gene significance (GS) measure was defined as (the absolute value of) the correlation between gene and external traits. Genes with highest MM and highest GS in modules of interest were natural candidates for further research [21]. Thus, the intramodular hub genes were chosen by external traits based GS > 0.2 and MM > 0.6 with a threshold of *P*-value < 0.05 [17]. The gene–gene interaction network was constructed and visualized using Cytoscape software package [22] and molecular complex detection (MCODE) [23] was used to analyze the most notable clustering module. MCODE score > 6 was a threshold for next analysis.

### Subjects

All of the two groups of study population were obese, including 424 unrelated participants of normals (128 males, 30.19% and 296 females, 69.81%) and 433 unrelated subjects of dyslipidemia (141 males, 32.56% and 292 females, 67.44%) were recruited from hospitalized patients in the First Affiliated Hospital, Guangxi Medical University [24]. The participants’ age ranged from 18 to 80 years with a mean age of 55.31 ± 10.52 years in normal and 55.87 ± 11.13 years in dyslipidemic groups; respectively. The gender ratio and age distribution were matched between the two groups. All participants were essentially healthy with no history of coronary artery disease, stroke, diabetes, hyper- or hypo-thyroids, and chronic renal disease. They were free from medications known to affect lipid profiles.

### Epidemiological survey

The epidemiological survey was carried out using internationally standardized method, following a common protocol [25]. Cigarette smoking status was categorized into groups of cigarettes smoker and non-smoker. Alcohol consumption was categorized into groups of alcohol drinker and non-drinker [26]. Several parameters such as blood pressure, height, weight and waist circumference (WC) were measured, while BMI (kg/m^2^) was calculated.

### Biochemical measurements

Venous blood samples were obtained from all subjects after at least 12 h of fasting. The levels of serum total cholesterol (TC), triglyceride (TG), high-density lipoprotein cholesterol (HDL-C), and low-density lipoprotein cholesterol (LDL-C) in samples were determined by enzymatic methods with commercially available kits, Tcho-1, TG-LH. Cholestest HDL, and Cholestest LDL, respectively. Serum apolipoprotein (Apo) A1 and ApoB levels were detected by the immunoturbidimetric immunoassay. All determinations were performed with an autoanalyzer in the Clinical Science Experiment Center of the First Affiliated Hospital, Guangxi Medical University [27].

### Diagnostic criteria

The normal values of serum TC, TG, HDL-C, LDL-C, ApoA1, ApoB levels and the ApoA1/ApoB ratio in our Clinical Science Experiment Center were 3.10–5.17, 0.56–1.70, 0.91–1.81, 2.70–3.20 mmol/L, 1.00–1.78, 0.63–1.14 g/L, and 1.00–2.50; respectively. Hypertension was diagnosed according to the criteria from the 1999 World Health Organization-International Society of Hypertension Guidelines for the management of hypertension [28]. The diagnostic criteria of overweight and obesity were according to the Cooperative Meta-analysis Group of China Obesity Task Force. Normal weight, overweight and obesity were defined as a BMI < 24, 24–28 and > 28 kg/m^2^, respectively [29]. Dyslipidemia was defined according to World Health Organization criteria: TG ≥ 1.7 mmol/L and HDL-C < 0.9 mmol/L for men or < 1.0 mmol/L for women. Diabetes was defined as a fasting plasma glucose ≥ 7.0 mmol/L or 2 h postprandial plasma glucose ≥ 11.1 mmol/L or as having been previously diagnosed with diabetes and receiving therapy [30].

### Bioinformatic analysis of miRNAs binding to SNP and linkage disequilibrium analysis

Bioinformatic software (http://bioinfo.life.hust.edu.cn/miRNASNP2/) was used to detect the candidate SNPs which could affect *COL1A1* regulation via miRNAs [31].

### RNA isolation

Fasting blood samples (5 mL) were collected in EDTA and separated by centrifugation at 3000*g* for 15 min. Total RNA containing miRNAs was isolated from plasma using the miRNeasy serum/plasma kit (TIANGEN: catalog number DP503, China). The homogenate was incubated for 5 min at room temperature, 25 fmol of synthetic cel-miR-39 (TIANGEN; catalog number: CD200-01, China) was spiked in. Subsequently, the RNA was extracted according to the manufacturer’s protocols. Total RNA was eluted in 30 µL of RNase-free water. RNA was reverse transcribed to cDNA with reverse transcriptase kit (TIANGEN; catalog number: KR211, China). The reaction system contained total RNA 2 µg, miRNA RT reaction buffer 10 µL, Enzyme Mix 2 µL, RNase-free water up to 20 µL. The mixture was incubated at 42 °C for 60 min, 95 °C for 3 min, and then held at 4 °C. A no-RT negative control was included in each experiment to ensure that PCR products were not due to contamination by genomic DNA.

### Reverse transcription (RT) and quantitative PCR (qPCR)

The quantification of 4 plasma miRNAs was measured by SYBR Green-based real-time PCR using a miScript SYBR Green PCR kit (TIANGEN; catalog number FP411, China). The reaction contained 2 × miRcute Plus miRNA Premix 10 µL, 0.2 μL PCR Forward Primer, 0.4 μL PCR Reverse Primer, 3.0 μL cDNA, RNase-free water up to 20 µL. The reactions were incubated at 95 °C for 15 min, 94 °C for 20 s, 60 °C for 30 s, 72 °C for 34 s. All reactions were run in duplicate. The average of the Ct value was calculated after the PCRs were run in duplicate for each sample. The cel-miR-39 value from the duplicate was used as the internal control [32]. The relative expression of each miRNA after normalization to cel-miR-39 is displayed as 2^− [Ct (miRNA) − Ct (cel-miR-39)]^.

### Statistical analysis

All statistical analyses were performed using the statistical software package SPSS 21.0 (SPSS Inc. Chicago, IL, USA) and R software (version 3.3.3). A Chi square analysis was used to evaluate the difference of the rate between the groups. Continuous data were presented as mean ± SD. For those, that are normally distributed, whereas the medians and interquartile ranges for TG, which is not normally distributed. Comparisons between groups for continuous data were made using Mann–Whitney nonparametric tests. The heart-map of correlation models and Bioinformatic analysis was measured by R software (version 3.3.3). The receiver operating characteristic (ROC) curve analysis was performed with plasma miR-3659 and miR-151a-5p to distinguish between dyslipidemia and control groups. The AUC was estimated to assess the diagnostic performance of miR-3659 and miR-151a-5p. All tests were two-sided, and *P* < 0.05 was considered statistically significant.

## Results

### Data preprocessing

When the GSE66676 was analyzed, we can get 54560 expression probes separately from each gene expression profile. After data preprocessing, the expression matrices of 19938 genes were obtained from the 67 samples. All of the genes and the samples’ phenotype were shown in Additional file 1: Tables S1 and S2.

### Weighted gene co-expression networks

**Fig. 2:**
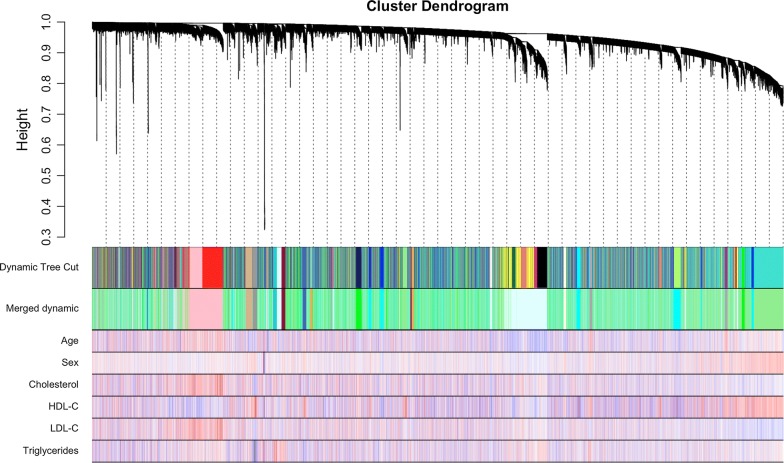
Clustering dendrogram of genes. Gene clustering tree (dendrogram) obtained by hierarchical clustering of adjacency-based dissimilarity. The colored row below the dendrogram indicates module membership identified by the dynamic tree cut method, together with assigned merged module colors and the original module colors. And, below is the phenotype. *HDL*-*C* high-density lipoprotein cholesterol, *LDL*-*C* low-density lipoprotein cholesterol

We selected soft-threshold β = 6 to construct gene modules using the WGCNA package (Additional file 2: Figure S1). After determining the soft threshold, all of genes were used to construct weighted gene co-expression networks. Then, we calculated the correlation matrix and adjacency matrix of the gene expression profile of the four lipid-profile groups, and then transformed them into a topological overlap matrix (TOM), and obtained a system clustering tree of genes on the basis of gene–gene non-ω similarity. Together with the TOM, we performed the hierarchical average linkage clustering method to identify the gene modules of each gene network (deepsplit = 2, cut height = 0.25). Six gene modules were recognized by the dynamic tree cut (Fig. 2).

### Finding module of interest and functional annotation

**Fig. 3:**
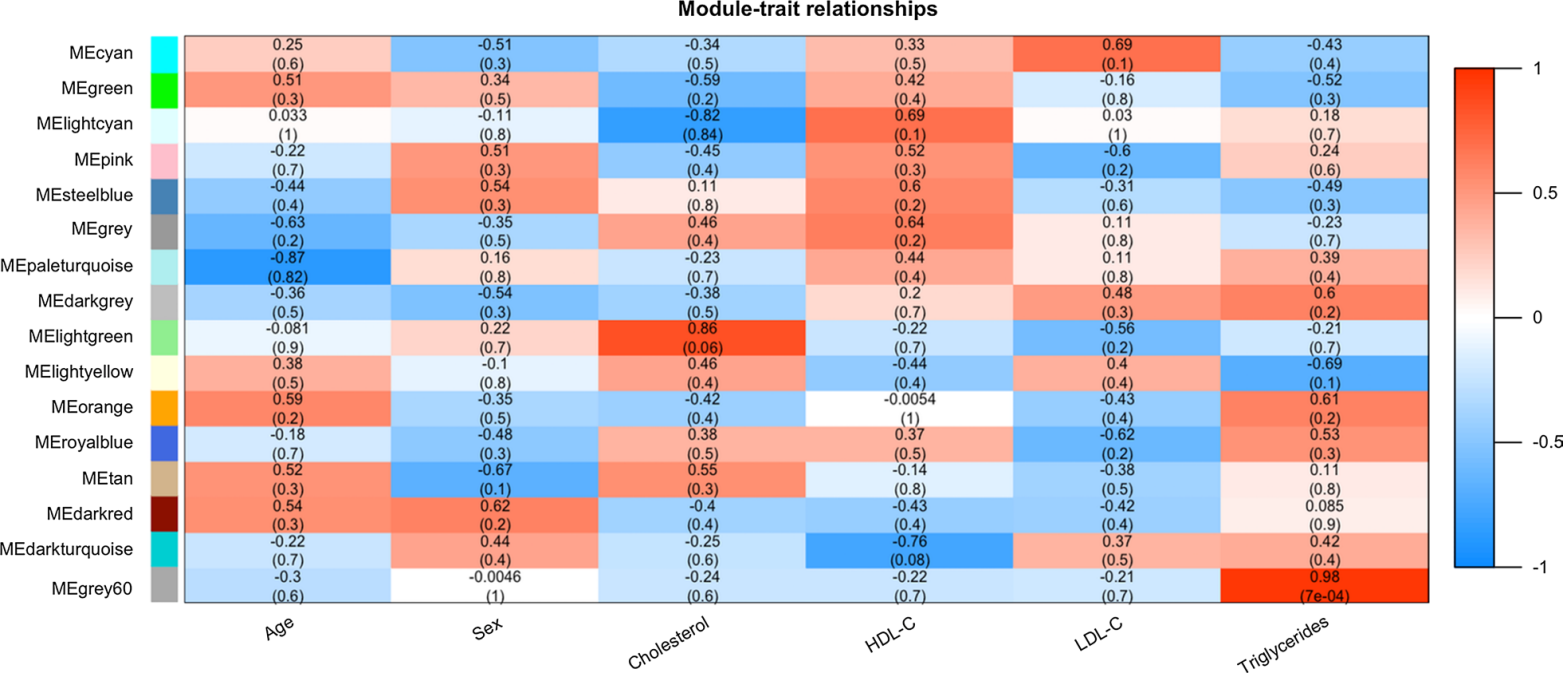
Module-feature associations. Each row corresponds to a module Eigengene and each column to a clinical feature. Each cell contains the corresponding correlation in the first line and the *P*-value in the second line. The table is color-coded by correlation according to the color legend

**Fig. 4:**
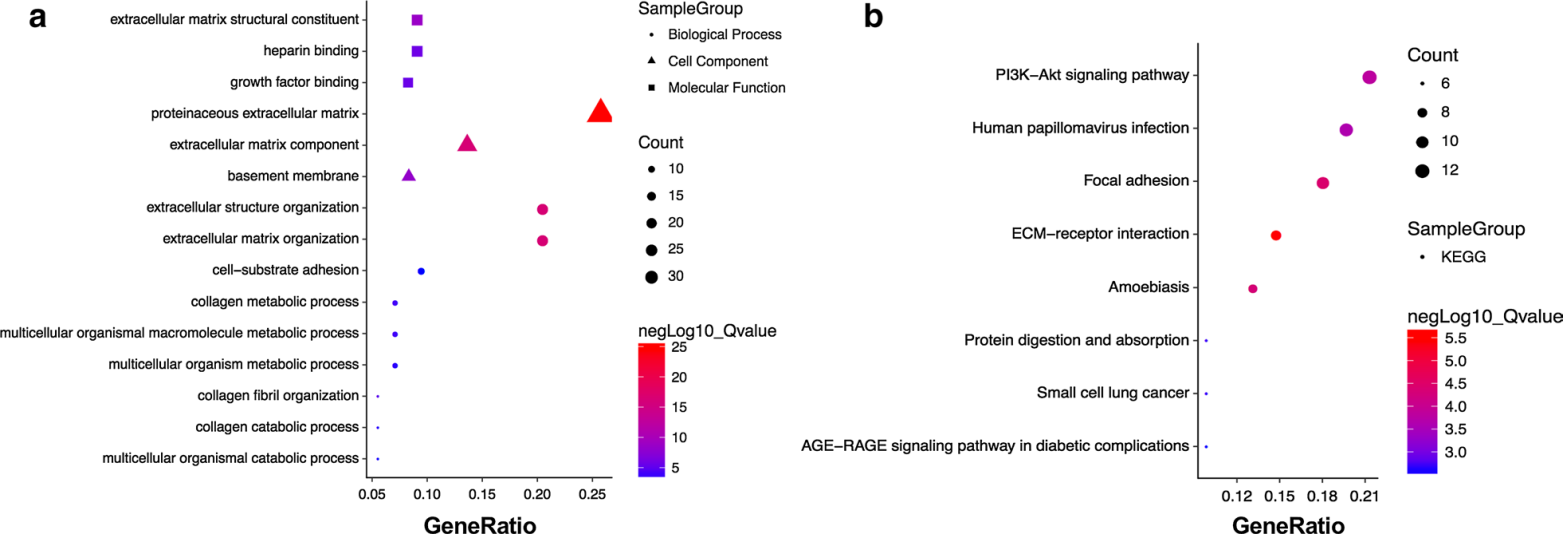
GO functional and KEGG pathway enrichment analyses for genes in the object module. The x-axis shows the ratio number of genes and the y-axis shows the GO and KEGG pathway terms. The − log_10_ (*P*-value) of each term is colored according to the legend. *GO* Gene Ontology annotation, *KEGG* the Kyoto Encyclopedia of Genes and Genomes pathway enrichment analyses

It is a hugely valued biological significance to find out modules most significantly associated with clinical features. The highest association in the Module-feature relationship was found in grey60 module and TG (*r*^*2*^ = 0.98, *P* = 7E-04), which was selected as module of interest and clinical feature to be studied in subsequent analyses (Fig. 3). The other modules without enough relationship or statistical significance for further consideration. In order to explore biological relevance of grey60 module, 139 genes which can be found in this module (Additional file 1: Table S3) were respective subjected to Gene Ontology (GO) functional and KEGG pathway enrichment analyses by R clusterProfiler package [20]. Biological processes, cell component, molecular function and KEGG pathway analysis of grey60 module were shown in Fig. 4. All of the databases were shown in Additional file 1: Tables S4 and S5.

### Protein–protein interaction (PPI) network construction and identify hub genes

**Fig. 5:**
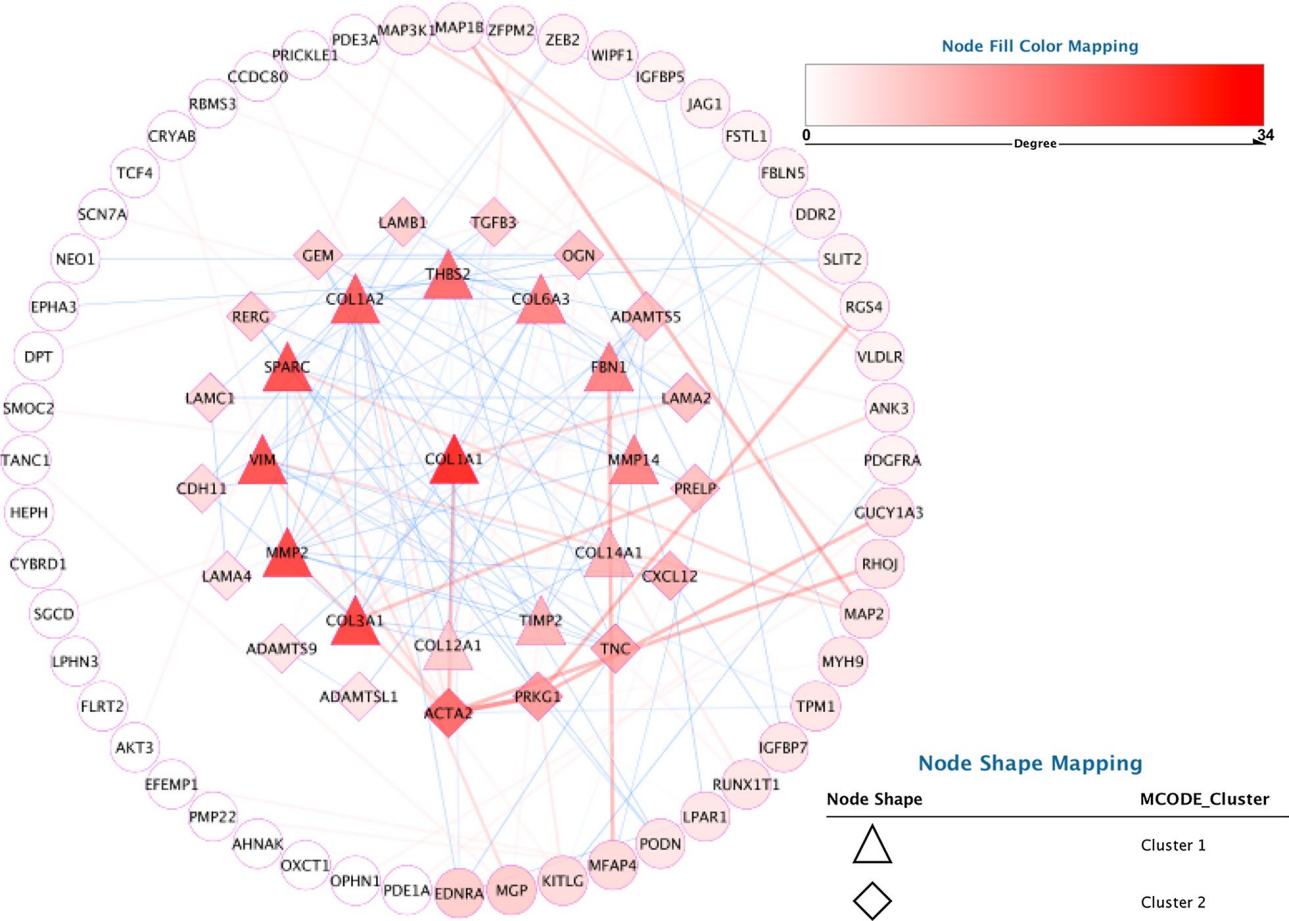
The protein–protein interaction analysis of the differentially expressed genes. Protein–protein interaction network of the module genes. Edge stands for the interaction between two genes. A degree was used for describing the importance of protein nodes in the network, red shows a high degree and blue presents a low degree. The significant two modules identified from the protein–protein interaction network shown with triangle (cluster 1) and diamond (cluster 2) using the molecular complex detection method with a score of > 6.0

When the STRING database [33] was analyzed, a total of 64 nodes and 179 protein pairs were got with a combined weight score > 0.25 in grey60 module (Fig. 5). After analysis in sub-module, only two modules with score > 6 were detected by MCODE. As shown in triangle cluster, *COL1A1* had the highest score (Degree = 34, MCODE = 10.25). We hypothesized that *COL1A1* as the hub gene was closely relevant to dyslipidemia occurs.

### Demographic and biochemical characteristics

Demographic, epidemiological and clinical characteristics of the 857 analyzed study subjects are summarized in Table 1. All of the subjects were obese. The levels of weight, WC, BMI, systolic blood pressure (SBP), diastolic blood pressure (DBP), pulse pressure (PP), serum glucose, TC, TG, LDL-C and the percentages of diabetes and hypertension were higher, whereas the HDL-C levels were lower in dyslipidemia group as compared with normals.

**Table 1:** Comparison of demographic, lifestyle characteristics and serum lipid levels between the normal and dyslipidemia groups in obese

Parameter	Normal	Dyslipidemia	Test-statistic	*P*
Number	424	433		
Male/female	128/296	141/292	0.561	0.454
Age (years)^a^	55.31 ± 10.52	55.87 ± 11.13	0.987	0.378
Height (cm)	156.13 ± 6.94	155.63 ± 7.02	1.496	0.192
Weight (kg)	52.83 ± 7.94	61.74 ± 10.64	25.439	1.73E–06
Body mass index (kg/m^2^)	29.49 ± 3.13	31.31 ± 4.54	31.224	2.56E–08
Waist circumference (cm)	74.23 ± 6.91	86.55 ± 9.47	22.321	3.11E–05
Smoking status [*n* (%)]^b^				
Non-smoker	306 (72.2)	325 (75.1)		
Smoker	118 (27.8)	108 (24.9)	0.920	0.337
Alcohol consumption [*n* (%)]				
Non-drinker	339 (80.1)	330 (76.2)		
Drinker	85 (19.9)	103 (23.8)	1.750	0.186
Systolic blood pressure (mmHg)	128.24 ± 18.18	136.47 ± 22.16	43.136	6.13E−012
Diastolic blood pressure (mmHg)	81.54 ± 10.16	86.49 ± 13.15	18.250	7.39E–05
Pulse pressure (mmHg)	49.64 ± 14.28	52.42 ± 17.59	28.317	3.63E−07
Glucose (mmol/L)	5.94 ± 1.83	7.15 ± 2.45	19.817	5.91E–05
Total cholesterol (mmol/L)	4.94 ± 1.13	5.14 ± 1.07	7.121	0.029
Triglyceride (mmol/L)^c^	1.49 (0.51)	1.78 (1.22)	8.441	0.021
HDL-C (mmol/L)	1.54 ± 0.49	1.06 ± 0.27	8.668	0.013
LDL-C (mmol/L)	2.84 ± 0.84	2.88 ± 0.79	9.497	0.007
ApoA1 (g/L)	1.33 ± 0.25	1.29 ± 0.27	0.364	0.558
ApoB (g/L)	0.82 ± 0.19	0.86 ± 0.20	1.492	0.233
ApoA1/ApoB	1.67 ± 0.50	1.66 ± 0.57	0.095	0.758
Diabetes [*n* (%)]	47 (11.0)	64 (14.9)	9.444	0.010
Hypertension [*n* (%)]	197 (46.4)	213 (49.3)	8.457	0.019

*HDL*-*C* high-density lipoprotein cholesterol, *LDL*-*C* low-density lipoprotein cholesterol, *Apo* apolipoprotein

^a^Continuous data were presented as mean ± SD and determined by two side *t*-test

^b^A Chi square analysis was used to evaluate the difference of the rate between the groups

^c^For those, that are normally distributed, whereas the medians and interquartile ranges for TG, was determined by the Wilcoxon–Mann–Whitney test

### Identification of *COL1A1* polymorphisms in 3′-UTR SNPs

**Fig. 6:**
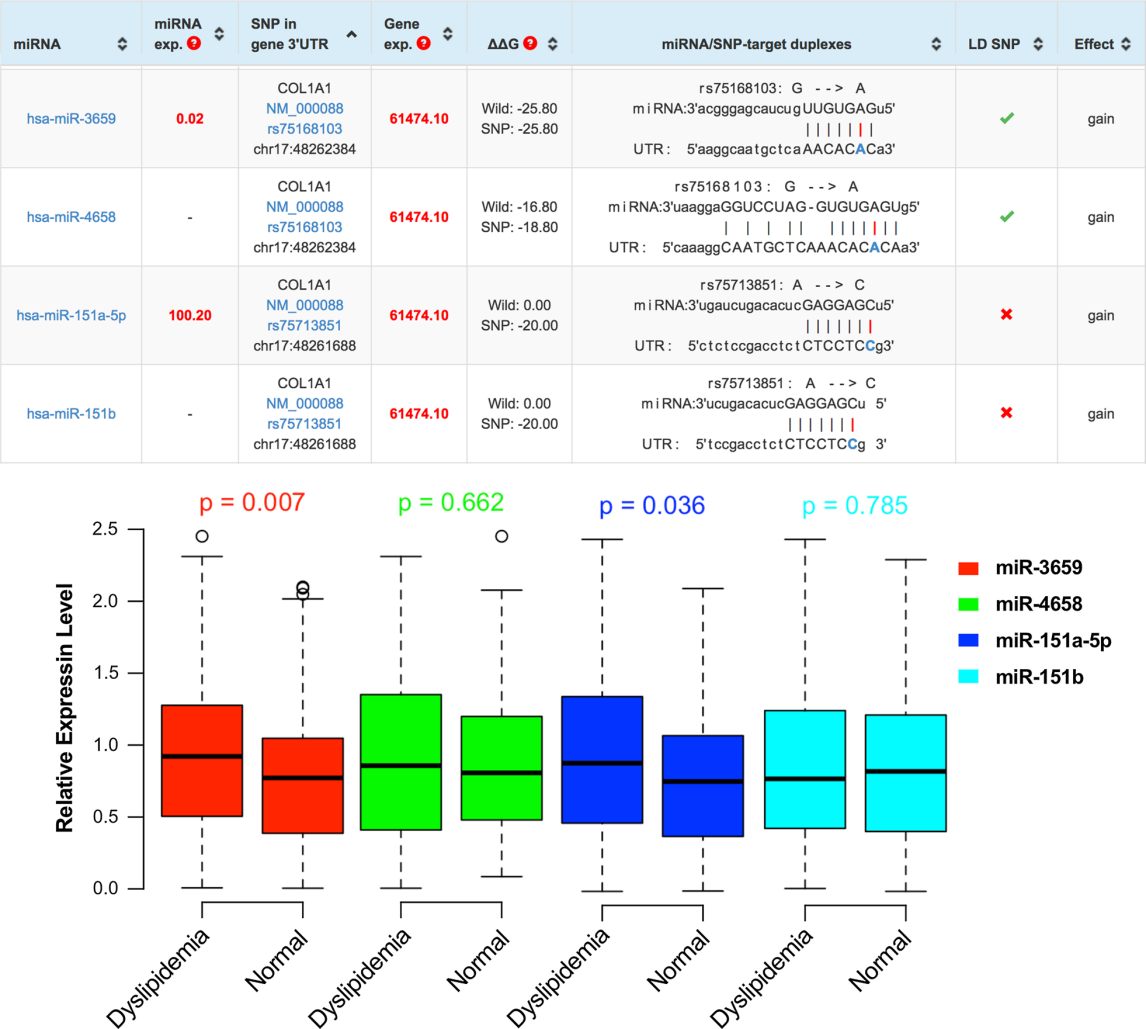
Binding of miRNA to *COL1A1* SNPs minor alleles and the relative expression level. On top of the figure shown bioinformatic analysis of potential miRNAs binding to *COL1A1* SNPs polymorphisms and below was the relative expression level of the four miRNAs between two groups

In this study, we mainly focused on the relationship of the SNPs in the *COL1A1* 3′-UTR to dyslipidemia risk and outcome. We first searched the GenBank of Single Nucleotide Polymorphism database (https://www.ncbi.nlm.nih.gov/snp) to identify potential *COL1A1* genetic variants in the 3′-UTR using the following parameters: Organism (Homo sapiens); Function Class (3′-UTR); Global MAF (0.01–0.1); Validation Status (by-1000 Genomes). We identified four *COL1A1* polymorphisms (Fig. 6).

### Expression level of four miRNAs between the two groups

As compared with those of healthy controls, the relative expression levels of circulating miR-3659 and miR-151a-5p in dyslipidemic patients were significantly increased (*P* < 0.05; Fig. 6).

### ROC curve for dyslipidemia

**Fig. 7:**
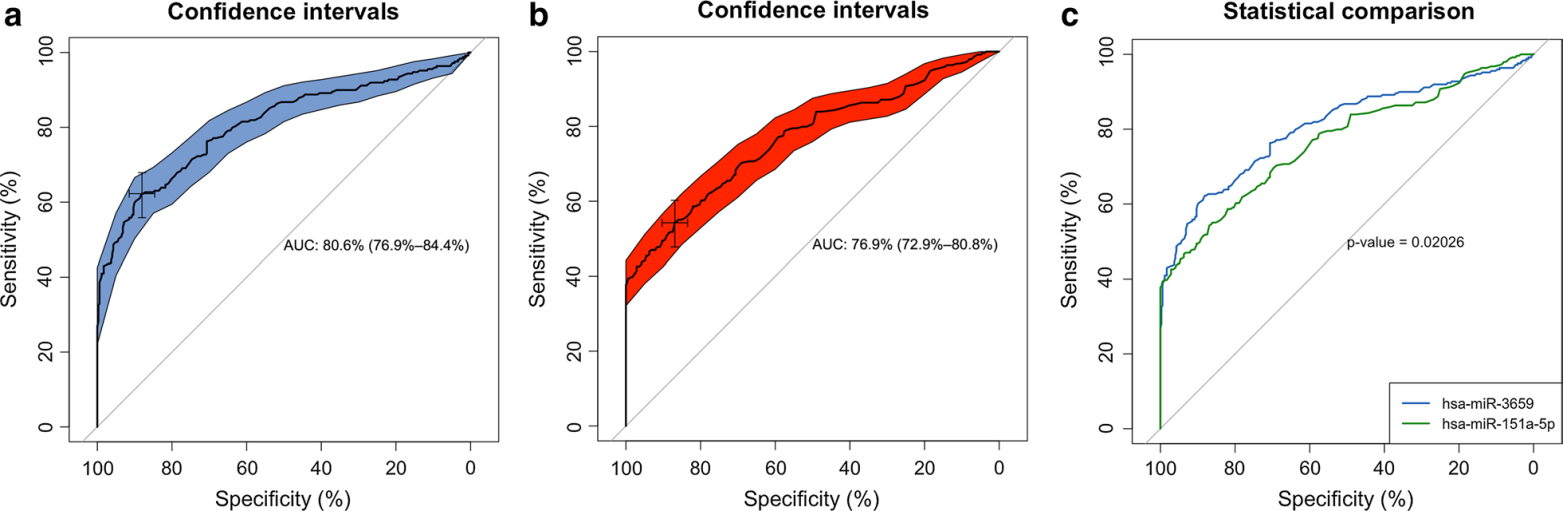
ROC curves for the predictive value of miR-3659 and miR-151a-5p regarding dyslipidemia. The ROC curves for the predictive values of miR-3659 and miR-151a-5p to identify dyslipidemic patients from healthy controls

We performed a ROC analysis to determine the predictive values of miR-3659 and miR-151a-5p for dyslipidemia. The AUCs of miR-3659 and miR-151a-5p were 0.806 (95% CI 0.769–0.844; *P* < 0.001) and 0.769 (95% CI 0.729-0.808; *P* < 0.001), respectively. This indicated that the diagnostic performance of the miR-3659 was superior to miR-151a-5p (*P* = 0.02026; Fig. 7).

## Discussion

Several recent reports showed that age, gender, smoking, obesity, dyslipidemia, lack of exercise, hypertension and diabetes mellitus are established risk factors for cardiovascular disease [34, 35]. With the remarkable improvement of social living standard, obesity has turned into a worldwide epidemic [36]. As a complex and multifactorial disease, lots of environmental and genetic factors can result in obesity and dyslipidemia [37, 38]. In the current study via WGCNA analysis, we have identified that *COL1A1* may modify serum lipid levels in obese patients.

Type 1 collagen is the main structural protein of bone and is encoded by two genes: *COL1A1* and *COL1A2*. The *COL1A1* is located in chromosome 17, region 17q21–22, and presents 51 exons. One of the most extensively studied polymorphisms is the so-called Sp1, which consists of the substitution of a guanine (G) by a thymine (T) in the first base of the first intron of the gene, which affects the binding site of the transcription factor Sp1 and thereby, the regulation of the gene transcription [39]. Besides this, a recent research found that *COL1A1* polymorphisms could modify blood lipid levels, especially TG [40]. Previous studies also showed that the typical dyslipidemia of obesity consists of increased TG and free fatty acid, decreased HDL-C with HDL dysfunction and normal or slightly increased LDL-C with increased small dense LDL [41]. Therefore, in obese, when the *COL1A1* expression changed, it may lead to serum TG increased and cause dyslipidemia.

MiRNAs, the endogenous and small noncoding RNAs, were found in lots of cell types and tissues especially the adipose tissue [42]. Besides, miRNAs may play an important role in the regulation of physiological and metabolic processes just as obesity, dyslipidemia, diabetes, aging, and others [43, 44]. Given their role in regulating transcriptional networks, miRNAs in adipose tissue might offer attractive biomarkers of adiposity as well as potential therapeutic targets for treating metabolic disorders [45].

Lots of evidences demonstrated that SNPs localized at miRNA binding sites (miRSNPs) could affect the binding of miRNAs to the target genes and in turn result in reduction or increase in translation of the target mRNA and altered susceptibility to disease [46]. In the current study, we used bioinformatic software to identified four miRNAs which can bind SNPs in 3′UTR in *COL1A1*, but only two miRNAs had statistical significance. After ROC curve analysis, we showed that miR-3659 in obese might be a potential biomarker for dyslipidemia diagnosis.

This study had several limitations. First, it did not report serum hormone levels which had potential impact on obesity and dyslipidemia. Second, it is a cross-sectional study that suggested hypotheses but failed to describe the relationship between the putative cause and effect. Third, it is a single-center study involving a small number of patients, and large-scale multicenter studies are necessary to verify our findings. Finally, the mechanisms of miR-3659 that regulated dyslipidemia in obese were not fully elucidated. The biological effects of microRNA in dyslipidemia need to be further determined via animal and cytology experiments in vitro.

## Conclusions

After comprehensive WGCNA bioinformatics analysis and verification, we found that miR-3659 was significantly elevated in dyslipidemia patients with obesity and exhibited a good predictive effect on the incidence of dyslipidemia in obesity.

## Additional files

**Additional file 1.** Additional tables.

**Additional file 2.** Additional figures.

## Abbreviations

GEO: Gene Expression Omnibus
WGCNA: weighted gene co-expression network analysis
GO: Gene Ontology annotation
KEGG: Kyoto Encyclopedia of Genes and Genomes pathway enrichment analyses
PPI: protein–protein interaction
MCODE: molecular complex detection
NCBI: National Center for Biotechnology Information
MM: module membership
Mes: module eigengenes
GS: gene significance
WC: waist circumference
SBP: systolic blood pressure
DBP: diastolic blood pressure
PP: pulse pressure
COL1A1: collagen type I alpha 1 chain
HDL-C: high-density lipoprotein cholesterol
LDL-C: low-density lipoprotein cholesterol
Apo: Apolipoprotein
TG: triglyceride
MetS: metabolic syndrome
BMI: body mass index
SNP: single nucleotide polymorphism
T2D: type 2 diabetes mellitus
TG: hypertriglyceridemia
