# Supplementary material for: Circulating miR-3659 may be a potential biomarker of dyslipidemia in patients with obesity
Source: J Transl Med. 2019 Jan 14;17:25. doi: 10.1186/s12967-019-1776-8 (PMC6332685; doi:10.1186/s12967-019-1776-8)
Supplement: Supplementary file 2 — Additional file 2. Additional figures. [file 12967_2019_1776_MOESM2_ESM.docx]

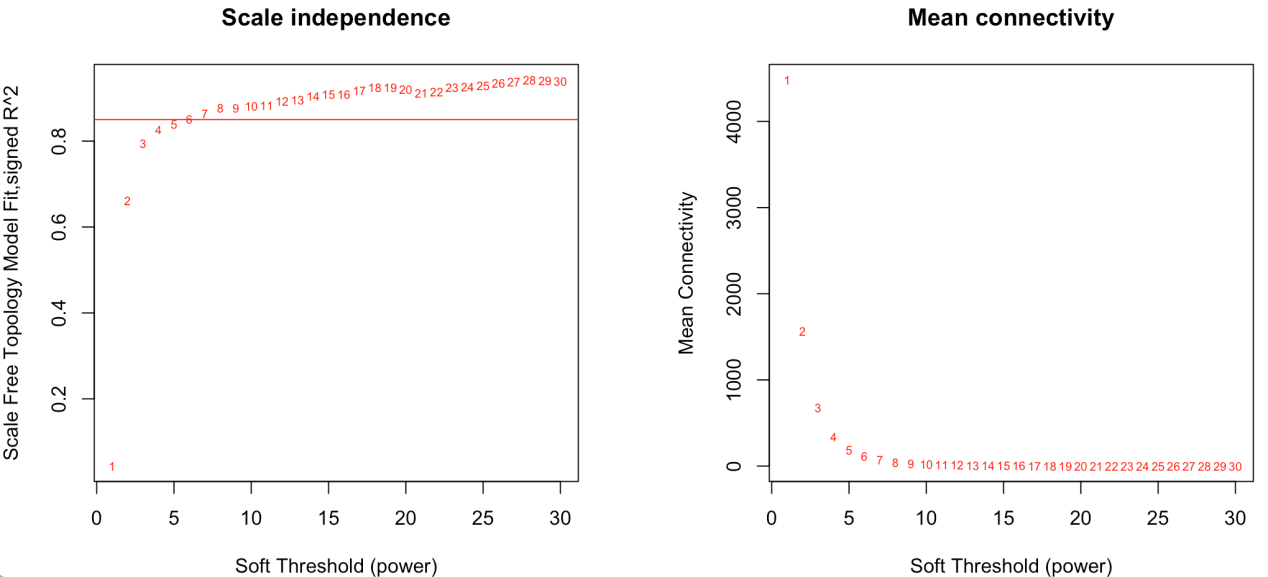


**Additional Figure S1: Analysis of network topology for various soft-thresholding powers.**

The left panel shows the scale-free fit index (y-axis) as a function of the soft-thresholding power (x-axis). The right panel displays the mean connectivity (degree, y-axis) as a function of the soft-thresholding power (x-axis).


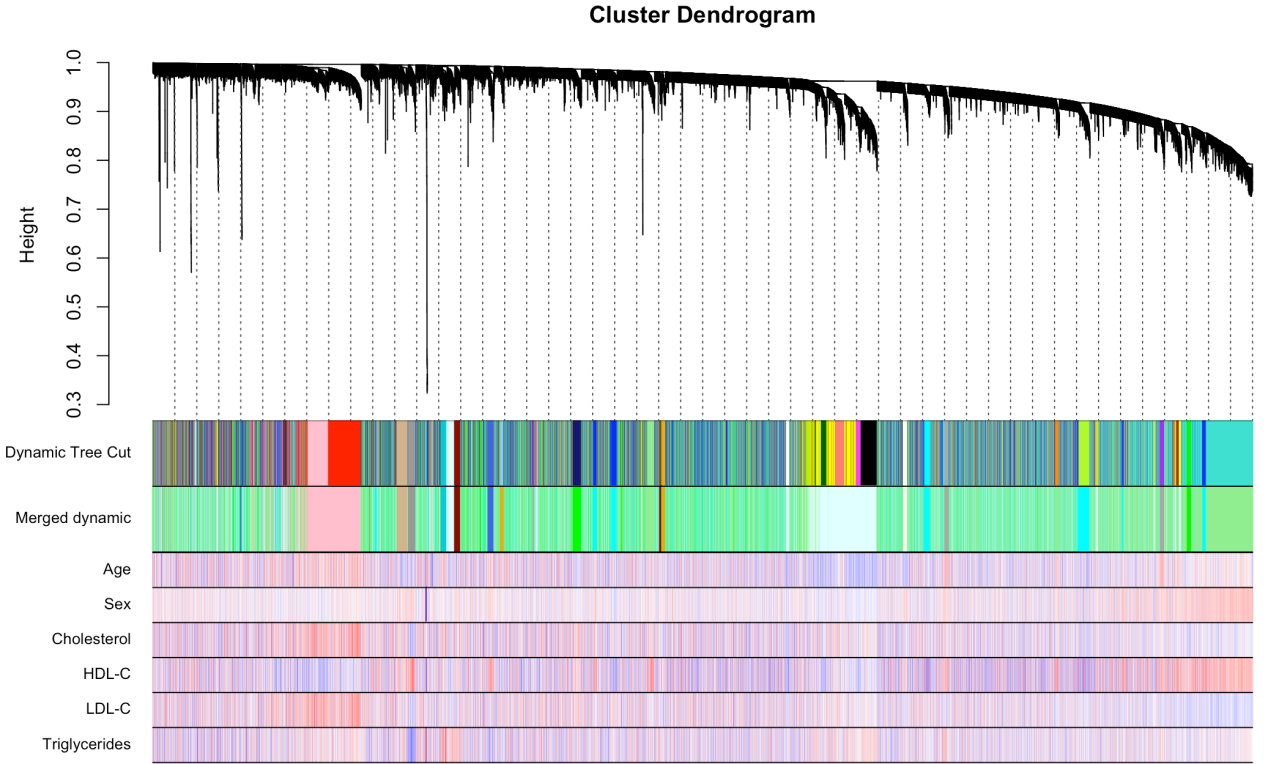


**Additional Figure S2: Clustering dendrogram of genes.**

Gene clustering tree (dendrogram) obtained by hierarchical clustering of adjacency-based dissimilarity. The colored row below the dendrogram indicates module membership identified by the dynamic tree cut method, together with assigned merged module colors and the original module colors. And, below is the phenotype.


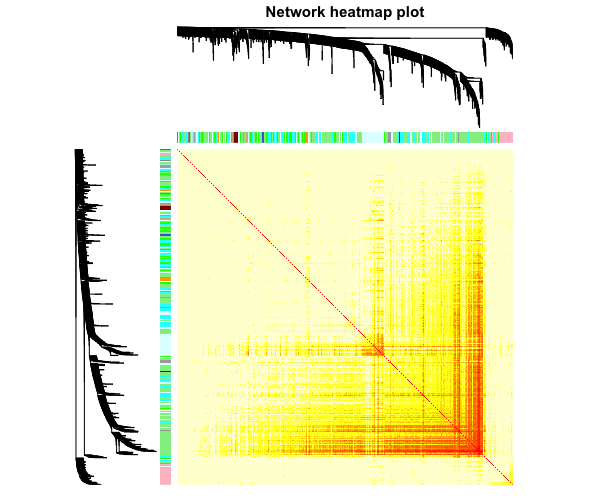


**Additional Figure S3:** **Heatmap plot of topological overlap in the gene network.**

In the heatmap, each row and column corresponds to a gene, light color denotes low topological overlap, and progressively darker red denotes higher topological overlap. Darker squares along the diagonal correspond to modules. The gene dendrogram and module assignment are shown along the left and top.


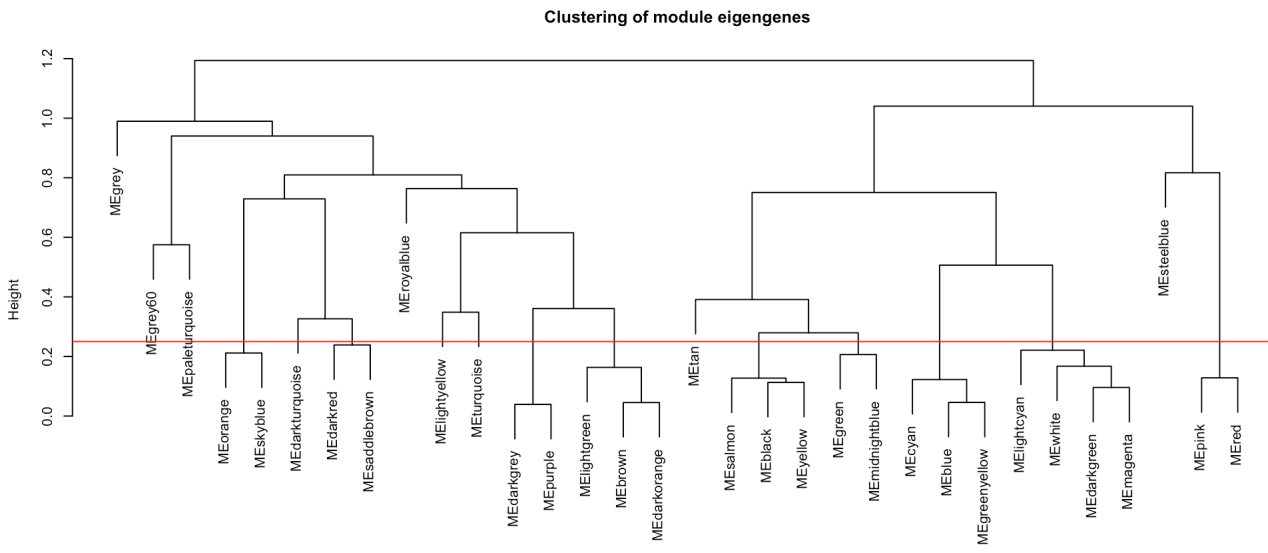


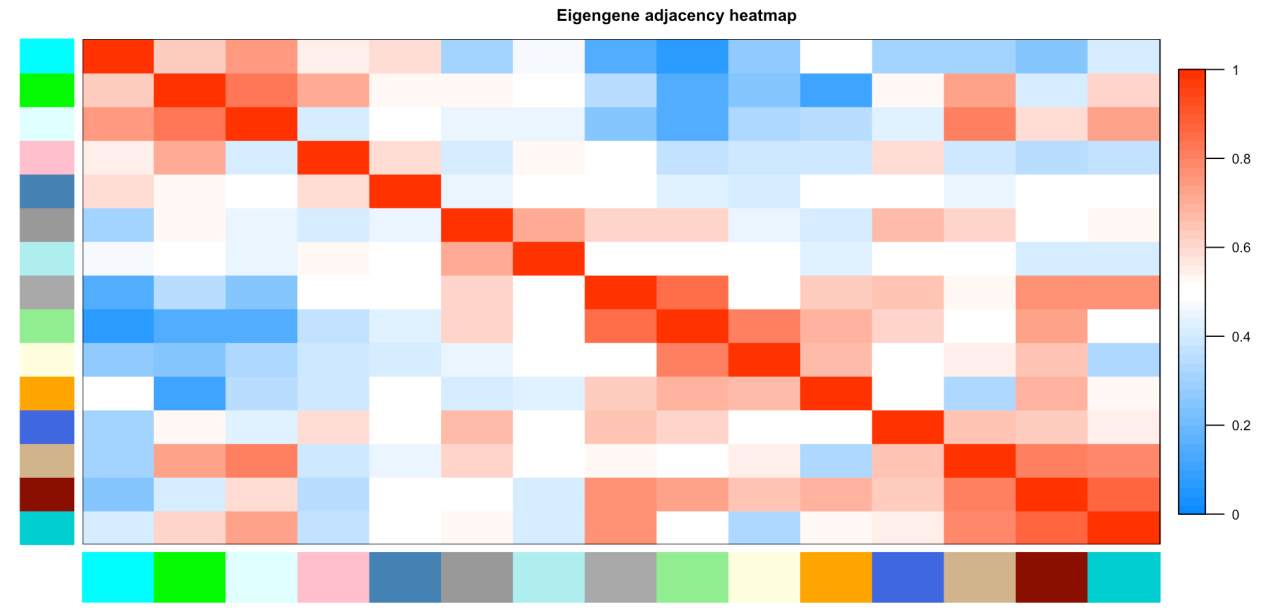


**Additional Figure S4:** **Relationships among modules.**

On top was hierarchical clustering of module eigengenes that summarize the modules found in the clustering analysis. Branches of the dendrogram (the meta-modules) group together eigengenes that are positively correlated. Below was heatmap plot of the adjacencies in the eigengene network. Each row and column in the heatmap corresponds to one module eigengene (labeled by color). In the heatmap, red represents high adjacency, while blue color represents low adjacency. Squares of red color along the diagonal are the meta-modules
